# The prevalence of people who inject drugs among those with HIV late presentation: a meta-analysis

**DOI:** 10.1186/s13011-022-00439-5

**Published:** 2022-02-10

**Authors:** Negin Farhadian, Behzad Karami Matin, Vahid Farnia, Mohammad Hossein Zamanian, Farid Najafi, Maryam Farhadian

**Affiliations:** 1 Substance Abuse Prevention Research Center, Health Institute, Kermanshah University of Medical Sciences, Kermanshah, Iran; 2 Research Center for Environmental Determinants of Health, Health Institute, Kermanshah University of Medical Sciences, Kermanshah, Iran; 3 Clinical Research Development Center, Imam Reza Hospital, Kermanshah University of Medical Sciences, Kermanshah, Iran; 4 Research Center for Health Sciences, Department of Biostatistics, School of Public Health, Hamadan University of Medical Sciences, Hamadan, Iran

**Keywords:** Late presentation, People who inject drugs, HIV, Transmission

## Abstract

**Background:**

One of the most important routes of HIV transmission is through injections of drugs, and this group, due to unawareness of their infection, causes the spread of HIV. The coexistence of other opportunistic infections and diseases with HIV among people who inject drugs (PWID) imposes healthcare costs and is associated with high morbidity/mortality rates. Early detection of HIV among PWID is essential to prevent and control the spread of the disease.

**Objectives:**

This study aimed to determine the prevalence of PWID among those with late presentation (LP).

**Methods:**

Three electronic databases of PubMed, Scopus, and Web of science were searched using appropriate keywords. Besides the prevalence data reported for PWID among LP, the other outcomes of interest were LP defined as having CD4 count < 350 cells/μL or HIV or advanced disease defined with CD4 count < 200 cells/μL or HIV at the time of diagnosis.

**Results:**

Of the 160 studies found, only eight met the inclusion criteria. Among those presented late, 36.5% were PWID (95% CI = 24.88–48.17). Compared with men who have sex with men (MSM), HIV-infected PWID had a higher risk of LP [OR = 1.51; 95% CI = 0.96–2.06].

**Conclusion:**

The results of this study show that HIV is diagnosed late in the majority of PWID when CD4 is less than 350 cells/μL. Targeted interventions/strategies are highly required to reduce LP among HIV-infected PWID.

## Introduction

Early detection of Acquired Immune Deficiency Syndrome allows infected people to use prophylaxis against opportunistic infections and beginning immediate treatment for secondary complications. People receiving treatment also benefit more social support and, more importantly, modify their behavior to prevent transmission of the virus to others [1]. Late presentation or delayed diagnosis of HIV in individuals is a significant barrier to new infections and related mortality [2]. Accordingly, access to medical care in the early stages of the disease is vital for both patient and the community [3, 4]. Despite efforts devoted to controlling and preventing HIV worldwide, 48% of newly HIV-infected patients are still diagnosed late, and even 27% are in advanced stages of the disease [5, 6]. HIV late presentation (LP) requires rapid clinical evaluations. It will increase mortality, the therapeutic response is poor, and even secondary toxicity to antiretroviral treatments is often higher. All of these cases impose high costs on the health care system and also speed up the transmission of the disease in communities [7, 8]. The main transmission categories of HIV are often men who have sex with men (MSM), heterosexuals with multiple partners, and people who inject drugs (PWID). The percentage of people who become infected with HIV due to drug injections has recently increased. In Italy and Spain, injecting drug is one of the most important risk factors for HIV, and in the United States, PWID is the second largest group of people living with HIV [9]. Due to the use of common ways of transmitting the disease, communities should give this group more attention. It has been found that severe conditions caused by other illnesses associated with HIV are more common in PWID, such as heart disease, liver and kidney disease, and malignancies. An HIV-infected person with comorbidities is up to 6 times more likely to die than a person without the other comorbid illness [10]. There is ample evidence that PWID does not have optimal access to antiviral therapies or show non-compliance to treatment and often tend to start treatment at more advanced stages of the disease [11, 12]. There are many public health benefits associated with the early detection of HIV in PWID. In fact, those who are aware of their HIV status are more likely to protect their partners and therefore prevent the further spreading of the virus [13]. It has been reported that PWID with HIV has lower survival compared to other groups of HIV/AIDS patients [14]. Although recent studies have examined the delay in the HIV presentation in various subgroups, including PWID, no review study has evaluated the prevalence of PWID in HIV LP. Accurate estimation of the prevalence of PWID in HIV LP is essential to determine epidemiological trends and to assess the benefits of prevention and treatment strategies.

## Methods

This meta-analysis study is designed based on the PRISMA rules [15]. Two authors performed the search process. Three databases of PubMed, Scopus and Web of science searched for relevant studies were published between February 1995 and February 2020 using the keywords: “HIV”, “AIDS”, “late”, “delay”, “presentation”, “diagnosis”, “testing”, “inject drugs”, and “drug use”. Study authors were not contacted, and manual search was not done. Two outcomes of interest are the prevalence of PWID with late presentation and the odds of PWID with LP comparing MSMs. The term “late presentation” and “advanced disease” are defined as having CD4 < 350 cells/μL and CD4 < 200 cells/μL, or clinically defined AIDS at enrollment, respectively [16].

This meta-analysis enrolled studies with the following conditions:

- All English observational studies have reported the prevalence of PWID with HIV late presentation among those with LP worldwide.

Studies that did not meet the inclusion criteria are the following:

- Studies that have provided other definitions for the LP and or have not categorized participants according to CD4 count.

All studies entered into Endnote reference manager software and duplicate papers were eliminated. Two authors independently reviewed the titles and abstracts as well as the full texts to determine related studies. If available, unadjusted reported odds of being PWID with LP as compared with MSMs were directly extracted from included records or calculated by using required data. Also, the proportion of PWID with LP to persons infected via hetero and homosexual contact as well as the proportion of PWID with LP compared to PWID with non-LP extracted. Two authors also evaluated the quality of the studies. Newcastle-Ottawa quality assessment forms for cohort and cross-sectional studies were used to assess the quality of studies. Statistical calculations of the results were performed using Stata software version 11. Random effects models were used to total effect size calculation, and the publication bias was estimated by the Begg’s and Egger’s regression tests.

## Results

**Fig. 1:**
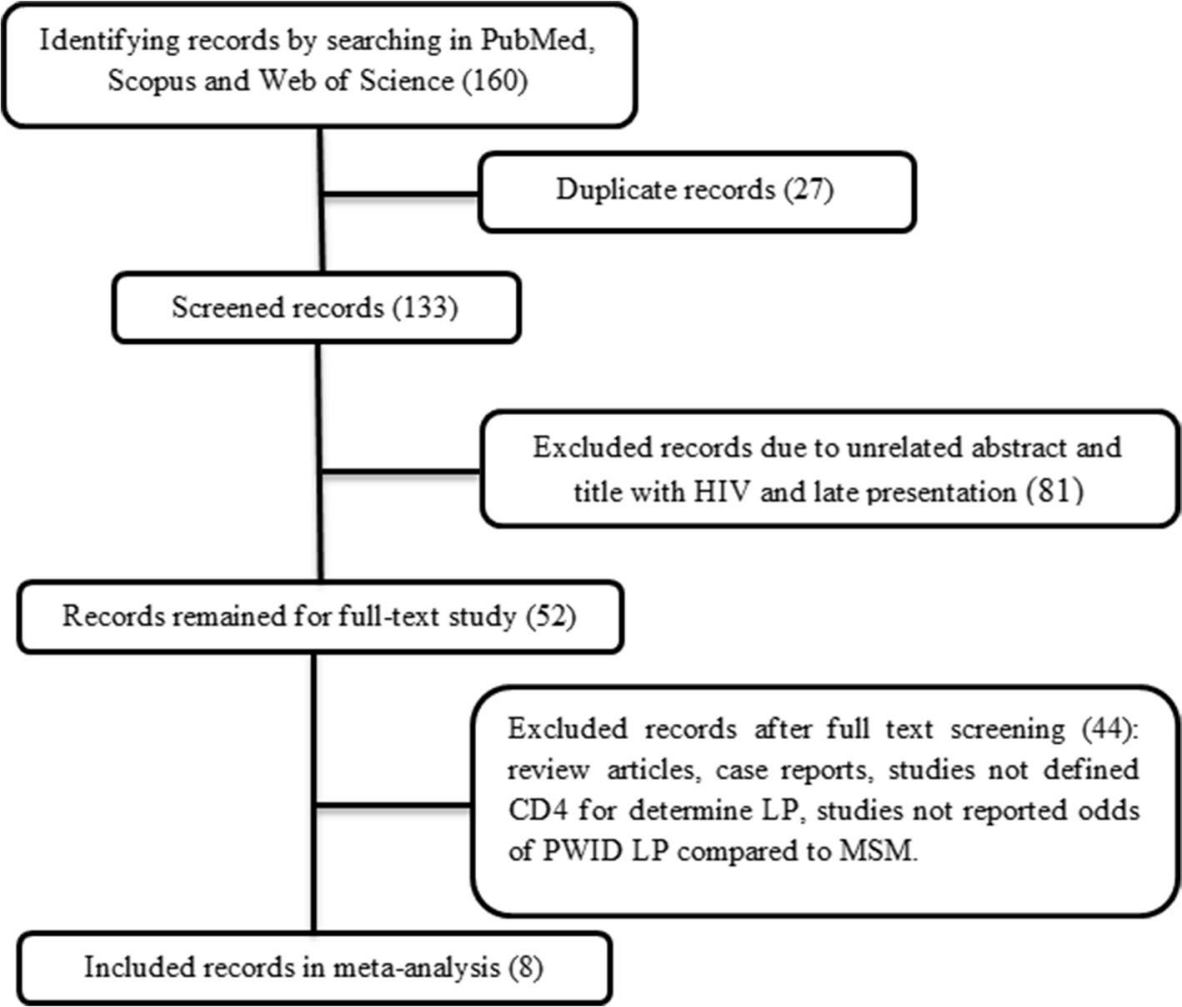
The search strategy

A search of the Scopus, PubMed, and Web of Science databases led to finding 160 articles, 10 of which ultimately had quantitative data to enter the meta-analysis. The process of selecting relevant studies is illustrated in Fig. 1. Among 10 included records, two cross-sectional and eight cohorts were found, thus meta-analysis was performed only on cohort studies. Detailed summaries of enrolled studies are in Table 1. According to the Newcastle-Ottawa checklists, all studies have good quality. Due to the different definitions used in studies to HIV late presentation, only the results of studies used that categorize LP based on CD4 count less than 350 and advanced disease based on CD4 count less than 200. Two responses were extracted from the records or calculated using the data provided.

**Table 1:** Detailed summaries of the enrolled studies

Authors, year	Region	Type of study	The proportion of PWID with LP among all HIV-infected PWID (95% CI)	The proportion of PWID with LP to all HIV-infected patients (95% CI)	The odds of LP in PWID compared to MSMs (95% CI)	The odds of advanced disease in PWID compared to MSMs(95% CI)	Quality score	References
Hu et al., 2019	China	cross-sectional	55 (52.4–58.4)	1.89 (1.74–2.04)	1.39 (1.2–1.67)	2.06 (1.73–2.45)	7*	[17]
Wójcik-Cichy et al., 2018	Poland	retrospective	80 (67.57–89.77)	17.37 (12.96–22.55)	3.49 (1.63–7.48)		8*	[18]
Brannstrom et al., 2015	Sweden	cohort	42.8 (24.46–62.82)	36.3 (18.9–62.5)	1.11 (0.49–2.49)		8*	[19]
Wilton et al., 2018	Canada	cohort	47.65 (39.41–55.98)	57.3 (45–71.7)	0.94 (0.66–1.34)		8*	[20]
Camoni et al. 2013	Italy	cohort	59.76 (54.23–65.11)	64.1 (55.7–73.3)	1.86 (1.46–1.67)		8*	[21]
Siwak et al., 2019	Poland	cross-sectional	66.42 (62.69–70.00)	19.87 (18.23–21.58)	2.02 (1.68–2.42)	2.68 (2.24–3.22)	8*	[22]
Sobrino-Vegas et al., 2012	Spain	cohort	26.64 (23.08–30.45)	12.31 (10.54–14.26)	0.72 (0.58–0.90)		8*	[23]
Op de Coul et al., 2014	Netherlands	cohort	78.77 (75.29–81.97)	42.5 (38.8–46.4)	2.53 (2.07–3.09)	2.9 (2.35–3.58)	8*	[24]
Vives et al., 2012	Spain	cohort	55.7 (51.78–59.74)	13.35 (12.06–14.71)	1.66 (1.36–2.03)		8*	[25]
Metallidis et al., 2012	Greece	retrospective	–	–	2.19 (0.98–4.9)	2.23 (1.02–4.86)	8*	[26]

### Prevalence of PWID among those with LP

**Fig. 2:**
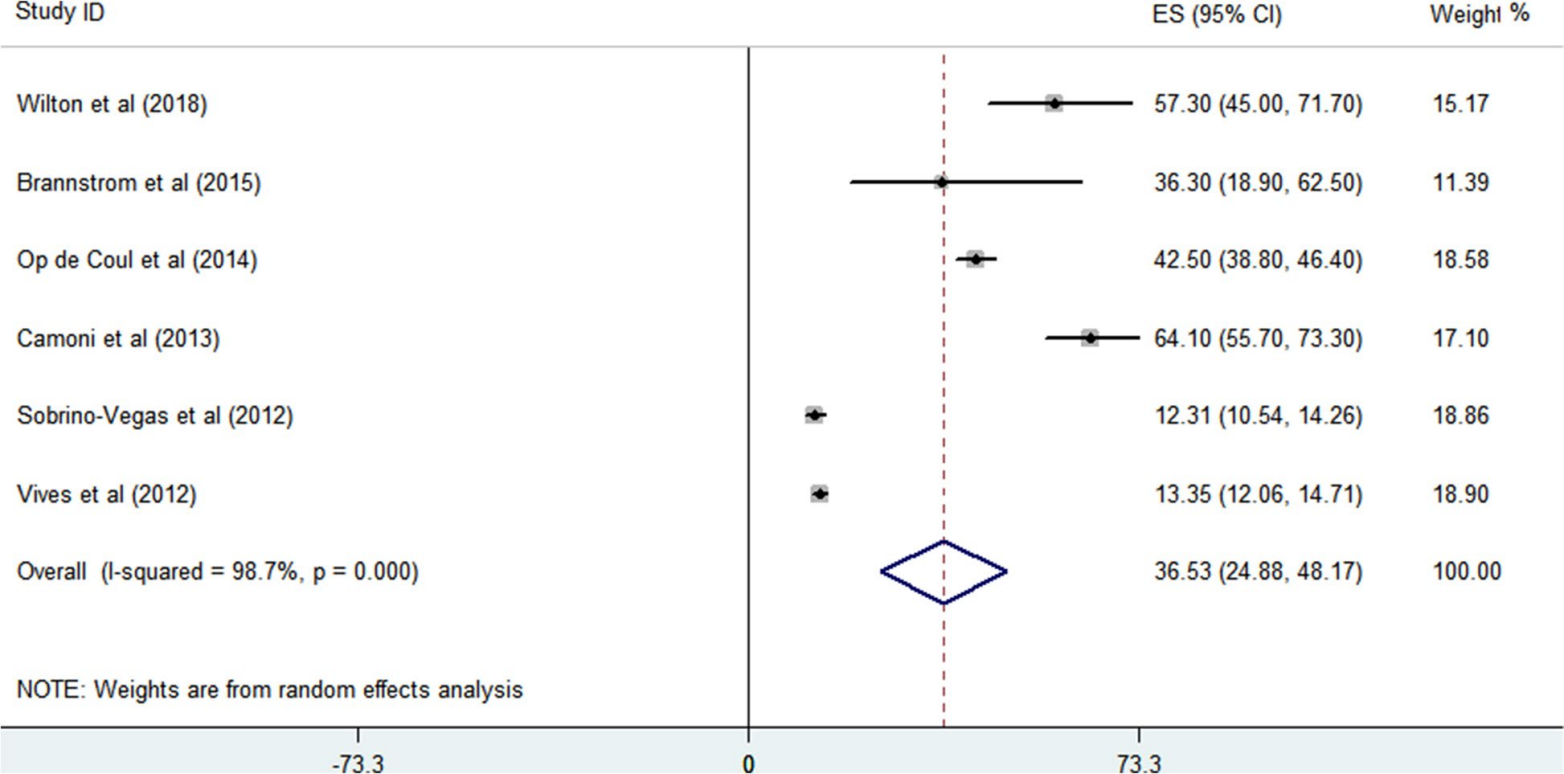
The proportion of PWID with LP to all HIV-infected patients

**Fig. 3:**
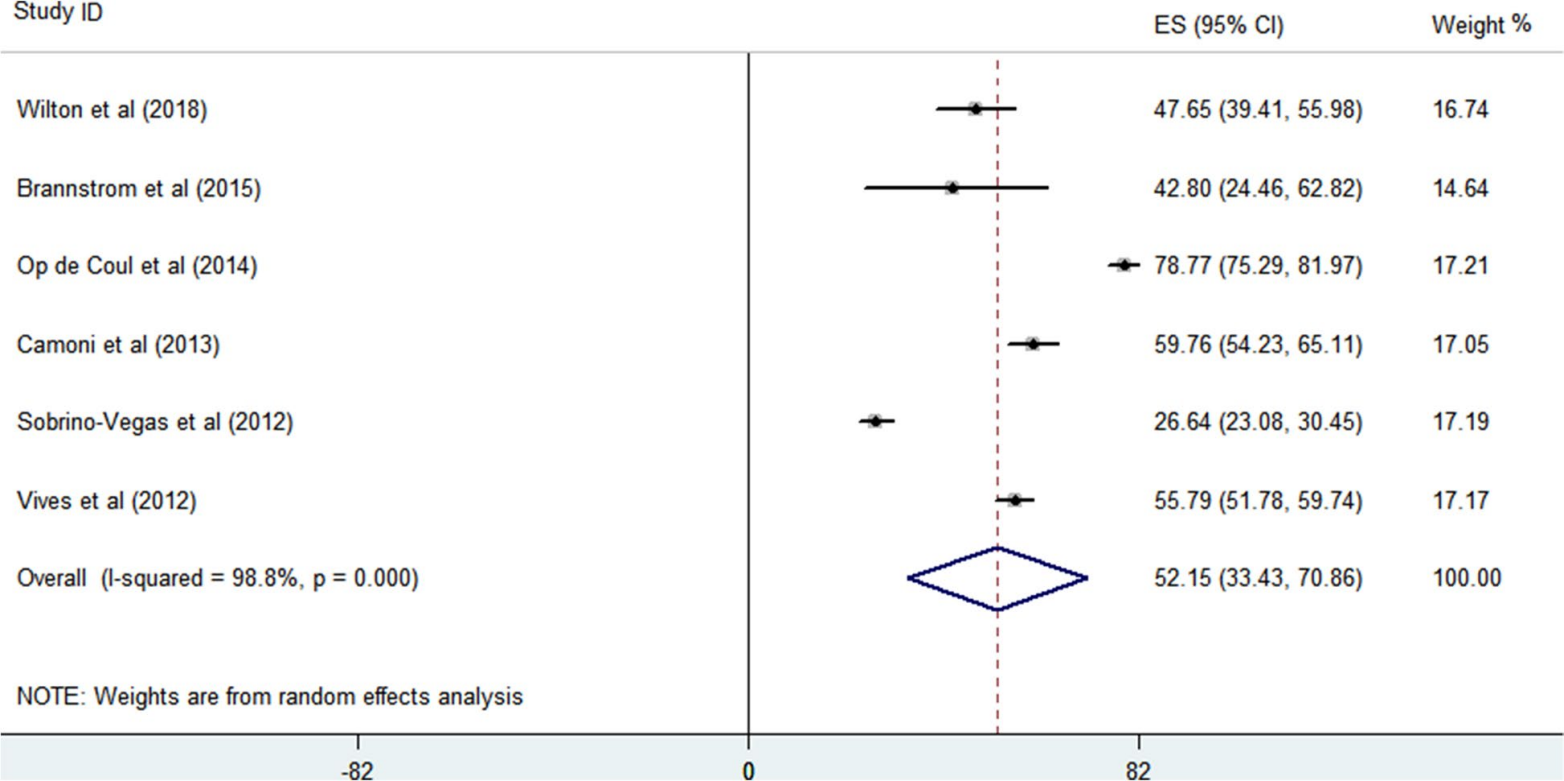
The proportion of PWID with LP among all PWID with HIV

The proportion of PWID with LP to all HIV-infected patients and the proportion of PWID with LP among all PWID with HIV were analyzed. Based on the findings of 6 enrolled studies, the proportion of PWID with LP to other HIV transmitted groups with LP is 36.53% (95% CI = 24.88–48.17) (Fig. 2). Begg’s and Egger’s regression tests *p*-values are 0.851 and 0.762, respectively. A large heterogeneity (98.7%) between the reported values for the prevalence of PWID with LP was detected in the included studies and reports varied from 12.31 to 64.10%. Also, the proportion of PWID with LP among all HIV-infected PWID is 52.15% (95% confidence interval = 33.43–70.86) (Fig. 3). Begg’s and Egger’s regression tests *p*-values are 0.851 and 0.090, respectively. The reported values for the proportion of PWID with LP among studies varied from 26.64 to 78.77% and large heterogeneity (98.8%) is observed between the results of different studies.

### The associations of LP in PWID compared to MSMs

**Fig. 4:**
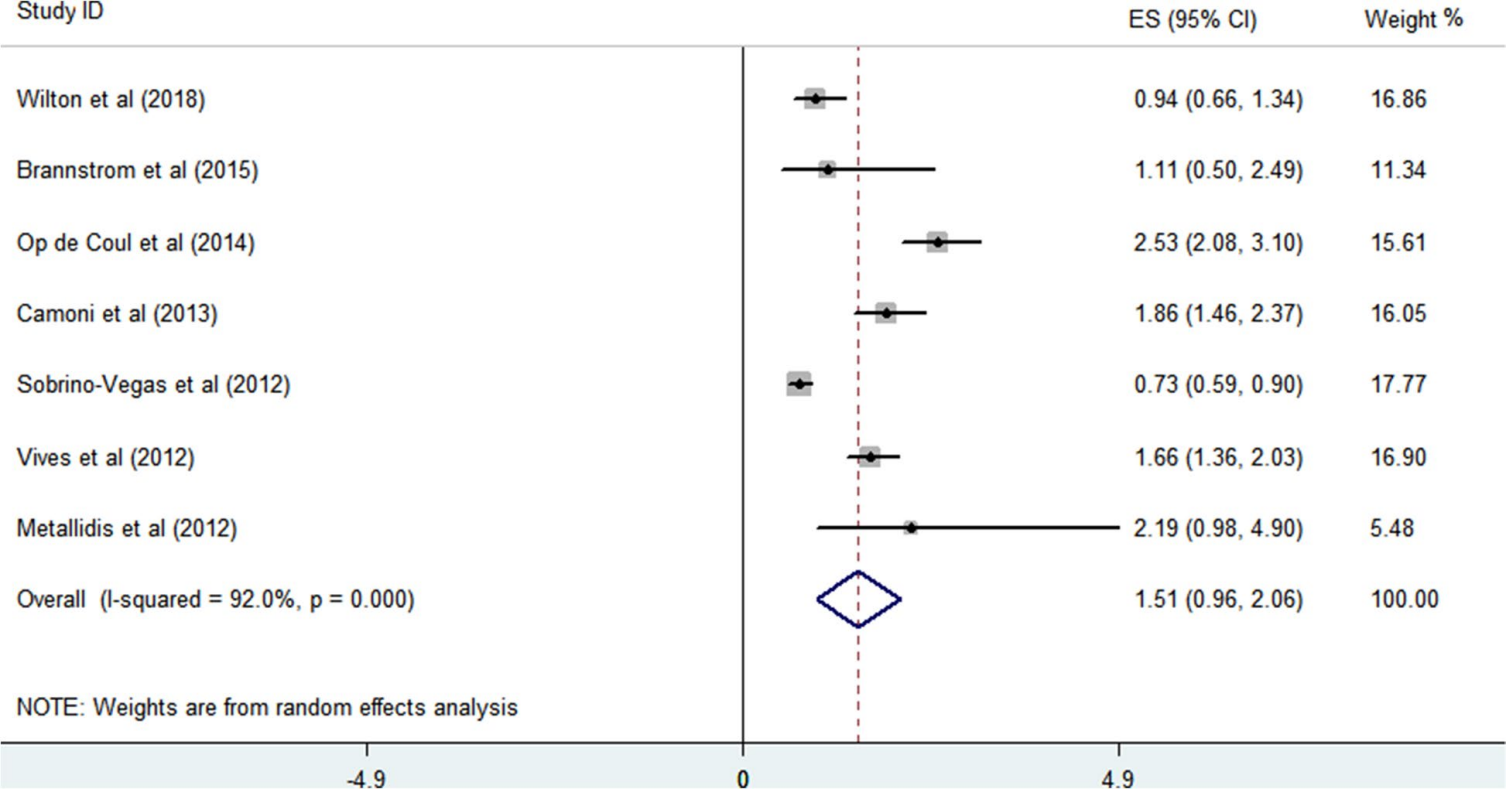
The associations (odds) of LP in HIV-infected PWID with LP than MSM

All included studies reported the associations of LP in PWID compared to MSM. Therefore, the values provided for the odds of LP in HIV-infected people through drug injection compared to HIV-infected MSMs were used. A meta-analysis of the results of seven studies defining LP based on a CD4 count of less than 350 showed a higher odds of LP in PWID than MSMs. Accordingly, HIV-infected PWID has a 1.51-fold chance of being LP compared to MSM (95% confidence interval = 0.96–2.06) (Fig. 4). No significant publication bias based on Begg’s and Egger’s regression *p*-values (0.652 and 0.756) was seen. The effect size reported in the included records varied from 0.73 to 2.53, and thus a significant variation in the result was evident (heterogeneity of 92%).

## Discussion

This meta-analysis study aims to investigate the prevalence of LP in the population of PWID with HIV compared to all other routes of HIV transmission. Compared to people who acquired HIV with other transmission categories, PWID are less likely to be diagnosed, counseled, and treated in the early stage of the disease. Patients who become infected with HIV through injecting drug use have a worse prognosis than those who have acquired HIV through other routes [27, 28]. The results of this meta-analysis study show that HIV is diagnosed late in a large number of PWID, and the probability of LP or diagnosis at advanced stages in this group is higher than MSM. MSM are less likely to be diagnosed with HIV late compared to PWID and heterosexuals. The reasons for this may be related to the early detection of HIV among MSM compared to other routes of transmission. Also, effective HIV testing strategies such as opt-out HIV testing at STI clinics and encouraging high-risk people to have regular HIV tests at regular intervals have improved the condition of this group. Establishing online facilities such as sending HIV test reminders to the individual and his or her partner has been helpful to this group and has led to earlier diagnosis of the disease [29–31]. In general, the lower rate of late diagnosis among MSM is due to more awareness and more frequent testing for this group, which leads to more asymptomatic individuals entering care.

Late presentation of HIV among PWID causes unintended transmission of the disease to others and the spread of infection, and due to delays in diagnosis, poorer treatment outcomes are seen in this group. In a study by Grigoryan et al. the survival of PWID was reported to be shorter compared to other high-risk HIV groups, and the reason for this was attributed to a delay in testing [32]. The mortality rate due to the higher incidence of opportunistic infections and complicated bacterial infections is higher in PWID diagnosed late [33]. Various factors such as age, gender, separation or divorce, immigration, low level of education and heterosexual contact are associated with LP. A meta-analytic study containing the results of 32 articles showed that men were 1.73 and 1.38 times more likely than women to have advanced HIV and LP [34]. However, some studies have reported conflicting results, and based on their results; women are more likely to be diagnosed late [35, 36]. Also, in studies, old age has been reported as one of the factors associated with LP [18, 37]. Inadequate public health interventions related to delays in testing, counseling, or poor communication with harm reduction programs are critical factors in the prevalence of HIV among PWID and increased LP in this high-risk group [33]. To evaluate people living with HIV as soon as possible, efforts should be made to raise awareness of the risk factors for HIV and to reduce missed testing opportunities. HIV testing for PWID can be done in a planned way at the time of their visit to treat addiction and syringe exchange programs. Having an understanding of the physiological as well as social reasons for late testing among PWID can help health care providers reduce barriers to testing in this group. Some studies have reported that PWID have less access to medical care and fewer visits to health clinics than those who have stopped injecting drug behaviors [38, 39]. This group uses fewer medical services and therefore needs more emergency services [1]. Less access of PWID to antiretroviral therapy has been reported in France [40]. Access and barriers to HIV testing vary among high-risk populations. For example, many PWID are reluctant to access health care systems when they are asymptomatic [25, 41]. It is necessary to consider various incentive strategies for regular periodic tests as well as enrolling and retaining in care along with addiction treatment and needle exchange programs for this group. Comprehensive prevention and control programs in different communities should be designed to support PWID. These programs lead to policies and practical intervention programs that can focus on HIV prevention, diagnosis, and CD4 cell counting tests among PWID. Further studies are required to identify factors associated with LP among PWID and to control the disease in this group.

Although we tried to extract the same outcomes from all the studies, there are limitations in generalizing the results of this meta-analysis. These studies have been conducted in different parts of the world, and access to health care services is not the same everywhere. For example, in some less developed areas, sicker patients with advanced stages of the disease are referred to healthcare centers. Also, due to the variety of considered factors related to late diagnosis in different studies, it is not possible to examine the impact of factors like the age of individuals, and socioeconomic factors on late presentation. Finally, the collection of retrospective information of patients in cohort studies is a challenge.

## Conclusion

The results of this meta-analysis show a high prevalence of late presentation of HIV in PWID. Late presentation leads to further spread of the disease as well as worse outcomes in this group. Development of interventions for significant factors associated with LP among HIV-infected PWID is recommended.

## Data Availability

Not applicable.

## Abbreviations

PWID: People who inject drugs
LP: Late presentation
MSM: Men who have sex with men
